# Force depression following a stretch‐shortening cycle depends on the amount of residual force enhancement established in the initial stretch phase

**DOI:** 10.14814/phy2.14188

**Published:** 2019-08-16

**Authors:** Rafael Fortuna, Tobias Goecking, Wolfgang Seiberl, Walter Herzog

**Affiliations:** ^1^ Human Performance Laboratory, Faculty of Kinesiology University of Calgary Calgary Canada; ^2^ Department of Biomechanics in Sports, Faculty of Sport and Health Sciences Technical University of Munich Munich Germany; ^3^ Institute of Sport Science, Department of Human Sciences Bundeswehr University Munich Munich Germany

**Keywords:** Cross‐bridge theory, eccentric contraction, electrical stimulation, force depression, residual force enhancement, titin

## Abstract

Studies on residual force enhancement (rFE) and residual force depression (rFD) of the muscle‐tendon unit (MTU) have typically been conducted independent of each other, with little information available on how stretch‐induced rFE affects the shortening phase and the steady‐state MTU isometric force at the end of stretch‐shortening cycles (SSCs). We showed previously that when rFE is kept constant, but the force at the end of the stretch is varied by changing the stretch speed, the steady‐state forces at the end of SSCs were the same. These results led to the hypothesis that the amount of rFE of the MTU established in the initial stretch phase of SSCs determines the steady‐state force following the shortening phase of SSCs. This study was aimed at testing this hypothesis. Steady‐state MTU isometric thumb adduction forces were measured for pure isometric contractions, following pure shortening contractions, following pure stretch contractions, and following SSCs with constant shortening speed and magnitude. However, two stretch magnitudes (30° and 10° thumb abduction) and stretch speeds (15°/sec and ~ 60°/sec, respectively) were chosen such that forces at the end of the stretch phase of the SSCs were the same, while rFE differed substantially. As hypothesized, the steady‐state isometric MTU forces following SSCs were positively related to the stretch‐magnitude dependent amount of rFE established in the stretch phase and were independent of the force reached at the end of the stretch phase in SSCs. Among many competing theories, these results can potentially be explained with the idea that there is a length‐specific engagement of a passive structural element at the initial length of muscle activation.

## Introduction

Human movement is characterized by a combination of lengthening and shortening of active muscles. In order to understand performance in human movements and everyday tasks, it seems essential to have insight into the processes underlying muscle force production in concentric, isometric, and eccentric contractions of the muscle‐tendon unit (MTU). Muscle stretch‐shortening cycles (SSCs) occur frequently during human movement (Komi 1984, 2000). In SSCs, active muscles undergo a stretching action that is immediately followed by a shortening contraction (Komi 2000). It has been shown that the force and work output during the shortening phase of SSCs exceeds those of exclusive shortening contractions (Cavagna et al. 1968; Bosco 1987). These findings suggest that muscle force production is history‐dependent and there appear to be two distinct phenomena that describe this history dependence (Abbott and Aubert 1952; Maréchal and Plaghki 1979; Edman et al. 1982; Joumaa et al. 2008a; Herzog 2014aa). The first of these phenomena is the long‐lasting enhancement in steady‐state isometric force observed following active  muscle stretching, typically referred to as residual force enhancement (rFE) (Abbott and Aubert 1952; Maréchal and Plaghki 1979; Edman et al. 1982); the second is the long‐lasting decrease in isometric steady‐state force observed following active muscle shortening, typically referred to as residual force depression (rFD) (Abbott and Aubert 1952; Maréchal and Plaghki 1979).

Studies on rFE and rFD of the MTU have typically been conducted independent of each other, with little information available on how rFE and rFD may affect force production in stretch‐shortening or shortening‐stretch cycles. The interaction of rFE and rFD in SSCs has been examined with conflicting results. Some earlier work on cat soleus suggested that the amount of rFD in SSCs was independent of preceding stretch, and thus not affected by rFE (Herzog and Leonard 2000; Lee et al. 2001). However, recent studies from the same, as well as other laboratories provide increasing evidence that stretch‐induced rFE contributes to increased force and work during SSCs, counteracting the development of rFD (Fukutani et al. 2015; Seiberl et al. 2015; Fortuna et al. 2017; Fukutani et al. 2017; Fortuna et al. 2018; Hahn and Riedel 2018).

In an effort to examine the relationship between rFD and rFE of the MTU, Fortuna et al. (2018) altered the force at the end of the stretch phase (by varying the stretch speed (Hill 1938; Katz 1939; Huxley 1957)), while leaving the amount of rFE constant (by maintaining the stretch magnitude (Katz 1939; Edman et al. 1982)). The shortening phase was kept identical for all experiments. They found that forces following these SSCs were the same, independent of the forces at the end of the stretch, suggesting that it was not the forces that reached in the stretch phase, but the amount of rFE that determined the steady‐state force in SSCs. This observation is in line with findings of previous studies (Hill 1938; Huxley 1957; Herzog and Leonard 2000) and led to the hypothesis that if shortening speed and amplitude are kept constant, rFD following SSCs directly depends on the amount of rFE established in the stretch phase, irrespective of the force at the end of the stretch or the work generated during shortening of the MTU.

The purpose of this study was to further test the hypothesis that the steady‐state isometric MTU forces following SSCs with fixed shortening parameters depend exclusively on the amount of rFE established in the stretch phase. A complementary experimental approach to the altered force at the end of the stretch experiments (Fortuna et al. 2018) was designed, where the forces at the end of MTU stretching were matched by adjusting stretch‐speed, while the amount of rFE produced in the stretch‐phase of SSCs was varied by changing the stretch‐amplitude (Abbott and Aubert 1952). We hypothesized that, even though forces at the end of stretch, and thus by definition, at the beginning of shortening, were matched, greater rFE would be established in the stretch phase, and would be associated with greater forces at the end of the SSCs, and thus, reduced force depression.

## Methods

### Participants

Twelve healthy subjects gave free informed consent to participate in this study (eight female and four male; age 27.6 ± 5.1 years; height 171 ± 8 cm; weight 67 ± 6 kg). All participants were free of neuromuscular disorders or any injury to the left hand. This study was approved by the Conjoint Ethics Committee of the University of Calgary and was in accordance with the guidelines established in the Declaration of Helsinki.

### Experimental setup

A custom‐designed dynamometer was used to measure thumb adduction forces and carpometacarpal angular displacements (Lee and Herzog 2002; Fortuna et al. 2012). Participants sat on an adjustable chair with the left arm slightly abducted and the elbow joint flexed at 90°. Movement of the wrist and fingers was restricted by using a reusable clinical cast (Ezeform, Rehabilitation Division, Smith & Nephew Inc., Germantown, USA) allowing only ab/adduction of the thumb. Two Velcro straps (one at the middle of the forearm and another in the area of the metacarpal bones) were used to secure the cast and to ensure the correct position of the arm during the experiment. A rotary stepper motor (Model TS42B‐P10, Parker Hannifin Corp., Cleveland, USA) was connected to an aluminum rod (1.5 cm diameter and 15 cm long) via gears (1:4 gears ratio). At the end of the rod an auxiliary piece for thumb placement and fixation was attached. The thumb pressed against the auxiliary piece, which was in line with the direction of thumb ab/adduction. The slightly supinated forearm led to movement of the thumb towards the third finger. The hand was positioned with the carpometacarpal joint aligned with the center of rotation of the motor to avoid slipping of the thumb during the experiment. Two pairs of calibrated strain gauges (Model CEA‐06‐0125UN‐350, Measurement Group Inc., Raleigh, USA) measured the thumb adduction forces. Thumb angle was measured using an analog encoder (Series 03 rotary transducer, Hohner Corp., UK). A digital controller (Model Gemini GT6‐L8 Digital stepper driver/controller, Parker Hannifin Corp., Cleveland, USA) controlled the rod. For every participant, the highest degree of thumb adduction before the lever arm touched the cast/hand was defined as the 0° reference angle for the following testing procedures.

### Electrical stimulation

The adductor pollicis muscle was stimulated electrically using two self‐adhering Ag–AgCl surface electrodes (2 × 3 cm) placed over the ulnar nerve. The cathode was placed 2 cm proximal to the metacarpal bones on the medial wrist and the anode 2 cm proximal to the cathode. The stimulation intensity was increased (frequency: 50 Hz, pulse duration: 0.5 *µ*sec) with a Grass S8000 stimulator (Astro Med Inc., Lingueil, Quebec, Canada) until approximately 50% of the maximal MTU isometric voluntary force (established separately) was reached. These stimulation parameters were then used for all contractions and lasted 7 sec. A 3‐min interval was given between stimuli to prevent fatigue. The term isometric MTU contraction is used to define that the MTU was kept at a constant length. However, such contractions, sometimes also referred to as fixed‐end contractions are often associated with substantial fascicle and sarcomere shortening (Hill 1938; Griffiths 1989; Griffiths 1991; Ito et al. 2017; Raiteri and Hahn 2019).

### Experimental Protocols 1 and 2

**Figure 1:**
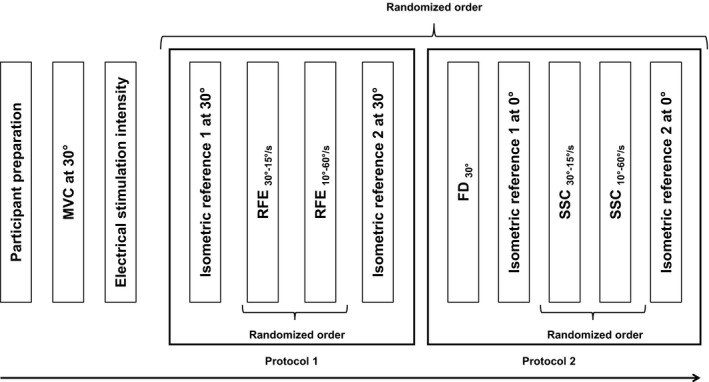
Experimental design for protocols 1 and 2. Following participant preparation, an isometric MVC at a 30° thumb abduction angle was performed and muscle stimulation intensity was adjusted so that a force of approximately 50% of the MVC was achieved. Protocol 1 started with an isometric reference contraction at a 30° abduction angle for 7 sec. Next, pure stretch (RFE) trials were performed under two conditions: (1) isometric pre‐activation at 0° and then stretched to 30° at 15°/sec (RFE_30°–15°/sec_), (2) isometric pre‐activation at 20° and then stretched to 30° at 60°/sec (RFE_10°–60°/sec_). Following the stretch phase, the muscle was held isometrically at 30° for 3.5 sec. Protocol 2 started with a pure shortening test (FD): isometric pre‐activation at 30° followed by shortening to 0° at 60°/sec (FD_30°_); followed by SSCs under two conditions: (1) isometric pre‐activation at 0° and then stretched to 30° at 15°/sec (SSC_30°–15°/sec_), (2) isometric pre‐activation at 20° and then stretched to 30° at 60°/sec (SSC_10°–60°/sec_). Following the stretch phase, the muscle was shortened to 0° at 60°/sec and held isometrically for 3.5 sec. At the end, a second isometric reference contraction was performed to assess muscle fatigue.

The experiment consisted of two protocols performed on two separate days. Protocol 1 comprised all residual force enhancement (rFE) trials and the corresponding isometric MTU reference contractions. Protocol 2 comprised force depression of the MTU (rFD), stretch‐shortening cycles (SSC), and the corresponding isometric MTU reference contractions. Two speeds and two magnitudes of MTU stretching were carefully combined to achieve the same force but different rFE at the end of the stretch phase of the SSCs. The order of the sessions, and the sequence of the conditions within each session, were randomized (Fig. 1).

### Protocol 1

Participants were comfortably positioned with the forearm in a neutral position and the elbow flexed 90°. Next, participants were verbally encouraged to produce a single 3 sec maximum voluntary contraction (MVC) at a 30° thumb abduction angle. The peak value of the MVC was identified and used for further analysis, while the passive force was not taken into consideration. Following the MVC test and adjustment of the stimulation intensity to reach about 50% of the MVC force, participants underwent an electrically evoked isometric MTU contraction at 30° of thumb abduction for 7 sec. Next, the RFE trials were performed using two conditions: (1) the MTU was pre‐activated and held isometrically at 0° for 1.5 sec and then stretched to 30° at a speed of 15°/sec (RFE_30°–15°/sec_), (2) the MTU was pre‐activated and held isometrically at 20° for 3.34 sec and then stretched to 30° at 60°/sec (RFE_10°–60°/sec_). The MTU was then held isometrically at 30° for 3.5 sec for both conditions. If the forces at the end of the stretch were not within 10%, the stretch speed was adjusted in RFE_10°–60°/sec_ (ranging from 60 to 100°/sec) until they were within 10% of each other. A second isometric MTU contraction at 30° was performed to assess fatigue.

### Protocol 2

Subjects performed a MTU MVC at 30° thumb abduction angle and the stimulation intensity was adjusted such that the electrically activated muscle reached approximately 50% of the MVC. Pure force depression tests of the MTU (FD_30°_) were then conducted and consisted of a 3 sec isometric phase at 30° followed by shortening to 0° at 60°/sec for 0.5 sec and a subsequent isometric steady‐state phase at 0° for 3.5 sec. Next, an isometric MTU reference contraction at 0° was performed for 7 sec. Following the isometric MTU reference contraction for rFD, SSCs were performed using two conditions: (1) the MTU was pre‐activated and held isometrically at 0° for 1 sec and then stretched to 30° at a speed of 15°/sec (SSC_30°–15°/sec_), (2) the MTU was pre‐activated and held isometrically at 20° for 2.84 sec and then stretched to 30° at 60°/sec (SSC_10°–60°/sec_). The MTU was then shortened from 30° to 0° at 60°/sec for both stretch conditions and held isometrically for 3.5 sec. If forces at the end of the stretch phase were not within 10% of each other, the short stretch condition (SSC_10°–60°/sec_) was repeated with modified speeds of stretch (ranging from 60 to 100°/sec) until forces at the end of stretch matched within 10%. A second isometric MTU reference contraction at 0° was conducted for 7 sec to assess fatigue (Fig. 1).

### Data reduction and analysis

**Figure 2:**
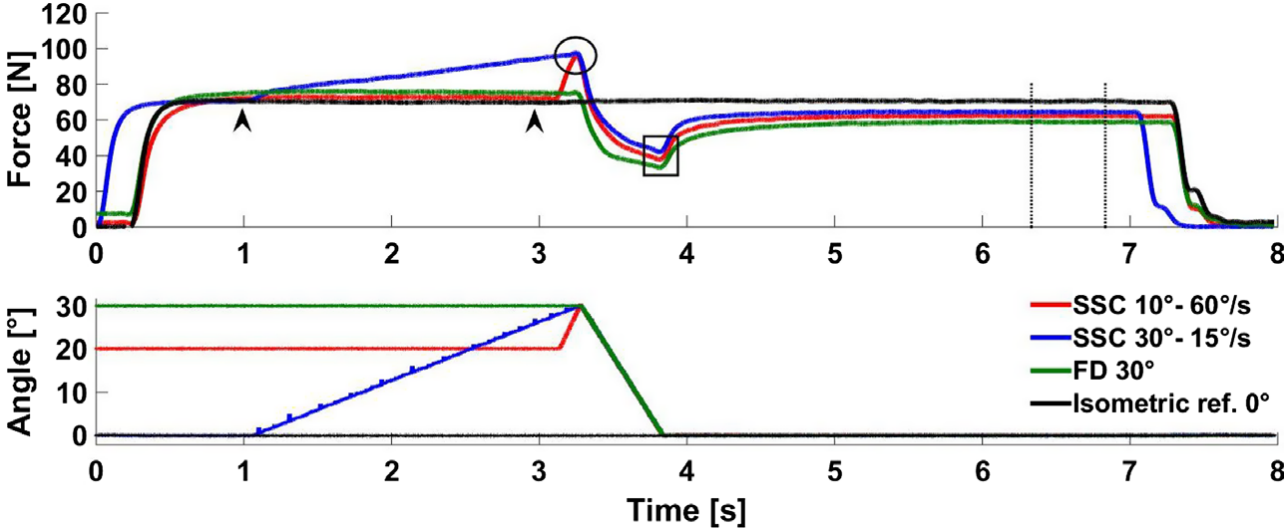
Representative thumb adduction force (top) and metacarpophalangeal joint displacement (bottom) as a function of time for protocol 2 for an isometric reference contraction at 0° (black line, Isometric ref. 0°), a pure shortening‐induced force depression (green line, FD_30°_), and SSCs of 30° thumb abduction amplitude at 15°/sec (blue line, SSC_30°–15°_) and 10° thumb abduction amplitude at 60°/sec (red line, SSC_10°–60°/sec_). The maximum force at the end of the stretch (e‐Str; ◯), the minimum force at the end of shortening (e‐Sho; □), and the average isometric force (500 msec) 2.5–3 sec after shortening or lengthening were assessed (vertical dotted lines). Forces were matched at the end of the stretch phase (±10%).

Data were sampled at 2000 Hz and collected via an analog‐to‐digital converter (Windaq, DATAQ Instruments Inc., Akron, USA). Force data were processed using a recursive Butterworth low pass filter to avoid the introduction of time lags with a cutoff frequency of 10 Hz. The MTU force at the end of stretch (e‐Str), minimum force at the end of shortening (e‐Sho), and the mean force (500 msec) 2.5–3 sec after the end of shortening (FD and SSC trials) or lengthening (RFE trials) in the steady‐state phase were used for statistical analysis (Fig. 2). Residual force enhancement of the MTU was expressed as the increase in the steady‐state isometric force relative to the isometric reference contraction at the corresponding thumb angle (30°). Force depression of the MTU for SSCs and pure rFD trials was expressed as the decrease in the steady‐state isometric MTU force relative to the isometric MTU reference contraction at the corresponding thumb angle (0°). Forces at the end of stretch (e‐Str) for the different conditions in Protocol 1 and 2 were considered matched if they were within ±10%. For pure MTU shortening (FD_30°_) and SSCs (SSC_30°–15°/sec_ and SSC_10°–60°/sec_), the mechanical work was calculated by integrating the force over the distance in the shortening phase. The mean force at the steady‐state for the rFD, SSC, and rFE trials were compared to the corresponding isometric MTU reference contractions to obtain the amount of rFD and rFE of the MTU.

Data were tested for normality (Shapiro–Wilk test) and either a one‐way repeated measures ANOVA (or non parametric Friedman test) with Bonferroni‐Holm (or Wilcoxon test) post hoc comparisons, or paired Students t‐tests were used to test for differences in the MTU force values at the end of the stretch, the minimum force at the end of the shortening, the mechanical work performed during shortening, and the steady‐state isometric forces 2.5–3 sec after lengthening or shortening. To attest for relations between the magnitude of rFD and mechanical work, as well as influences of stretch parameters on the steady‐state MTU isometric forces after rFE and SSCs with same stretch speed and magnitude, bivariate correlation analysis were applied.

Force redevelopment analysis following the MTU shortening phase was made using the commercial software MATLAB (R2014b, MathWorks, Inc. Natick, MA) and a double exponential function (Seiberl et al. (2015); Corr and Herzog 2016), as shown below:

(1) $$F_{\mathrm{re}}\left(t\right)=A_{s}\times {\text{e}}^{\left(k_{s}\times t\right)}+A_{f}+{\text{e}}^{\left(k_{f}\times t\right)}$$

In order to compare force redevelopment functions across tests and subjects, MTU forces were normalized between 0 (minimum force reached at the end of shortening) and 1 (steady‐state force reached after force redevelopment). *A_s_* and *A_f_* represent the amount of MTU force in the slow and fast phase of recovery, while *k_f_* and *k_s_* represent the corresponding rates of MTU force recovery. Statistical significance was set for all tests at *P* < 0.05.

## Results

### Protocol 1

Mean MVC at 30° thumb angle for assessment of 50%‐MVC stimulation intensity in Protocol 1 was 101.2 ± 18.0N. There was no statistical difference (*t*(11) = 0.076, *P* = 0.941) between the MTU isometric reference contraction at the beginning (48.5 ± 10.9N) and at the end (48.4 ± 11.4N) of protocol 1.

**Figure 3:**
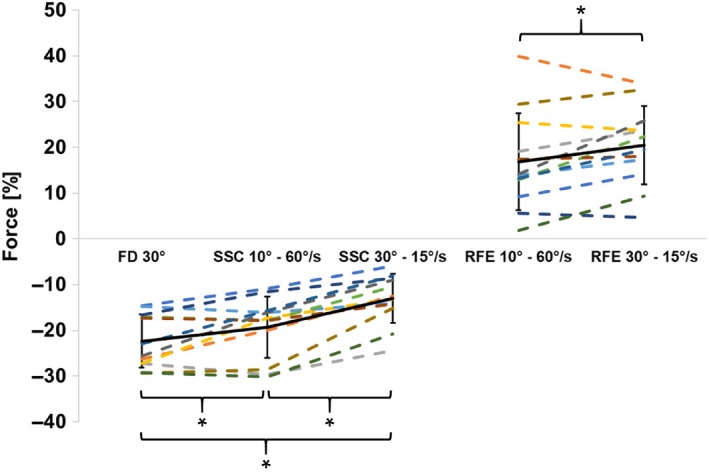
Mean (±SD) values of force depression for FD_30°_, SSC_10°–60°/sec_, and SSC_30°–15°/sec_ (protocol 2) and residual force enhancement for the RFE_10°–60°/sec_ and RFE_30°–15°/sec_ (protocol 1) normalized to the isometric reference contraction at the corresponding angle. All conditions in protocol 2 were significantly (*P* < 0.01) different from each other. FD30° showed the lowest minimum value of 22.4 ± 5.8 N, followed by SSC_10°–60°/sec_ and SSC_30°–15°/sec_ (19.3 ± 6.7 N and 13.0 ± 5.4 N, respectively). Additionally, rFE was statistically (*P* < 0.05) greater in RFE_30°–15°/sec_ (20.5 ± 8.6 N) compared to RFE_10°–60°/sec_ (16.9 ± 10.5 N).

MTU Forces at the end of stretch for RFE_10°–60°/sec_ (71.0 ± 11.6N) and RFE_30°–15°/sec_ (72.2 ± 12.7N) were not significantly different (*t* (11) = −1.251, *P* = 0.237) and, by design, matched within a range of ±10% for every participant (Table 1). MTU Isometric steady‐state forces varied significantly (*F* (2,22) = 57.56, *P* < 0.001) between the isometric reference and rFE contractions. Post‐hoc analysis revealed steady‐state MTU isometric force after stretch was significantly (*P* < 0.001) higher by 16.9 ± 10.5% for RFE_10°–60°/sec_ and by 20.4 ± 8.6% for RFE_30°–15°/sec_ compared to the isometric MTU reference contraction at the corresponding thumb angle (Fig. 3). Residual force enhancement of the MTU at the steady‐state following stretch was significantly (*P* < 0.05) greater for RFE_30°–15°/sec_ compared to RFE_10°–60°/sec_ (Fig. 3).

**Table 1:** Mean values (±SD) of force at the end of stretch (e‐Str), end of shortening (e‐Sho), and work for FD_30°_, SSC_10°–60°/sec_, SSC_30°–15°/sec_, RFE_10°–60°/sec_, RFE_30°–15°/sec_. (^*^compared to FD_30°_. ^†^compared to SSC_10°–60°/sec_).

Contraction condition	e‐Str [N]		e‐Sho [N]		Work [J]	
	Mean	SD	Mean	SD	Mean	SD
FD_30°_	–	–	16.6	7.8	1.51	0.41
SSC_10°–60°/sec_	79.5	14.5	20.2^*^	8.3	1.95^*^	0.46
SSC_30°–15°/sec_	82.0	13.9	24.9^*†^	8.7	2.17^*†^	0.48
RFE_10°–60°/sec_	71.0	11.6	–	–	–	–
RFE_30°–15°/sec_	72.2	12.7	–	–	–	–

### Protocol 2

Mean MVC at 30° thumb angle for assessment of 50%‐MVC stimulation intensity in Protocol 1 was 112.2 ± 20.3N. The mean electrically evoked MTU isometric force before shortening in the pure shortening contraction FD_30°_ at the corresponding 30° thumb angle of the MVC was 58.5 ± 12.4N, representing 52% of the participants MVC. There was a statistically significant (*t*(11) = 4.016, *P* = 0.002) 2.3% decrease in the maximal isometric reference force from before (51.6 ± 11.8N) to after (50.4 ± 11.5N) protocol 2.

**Figure 4:**
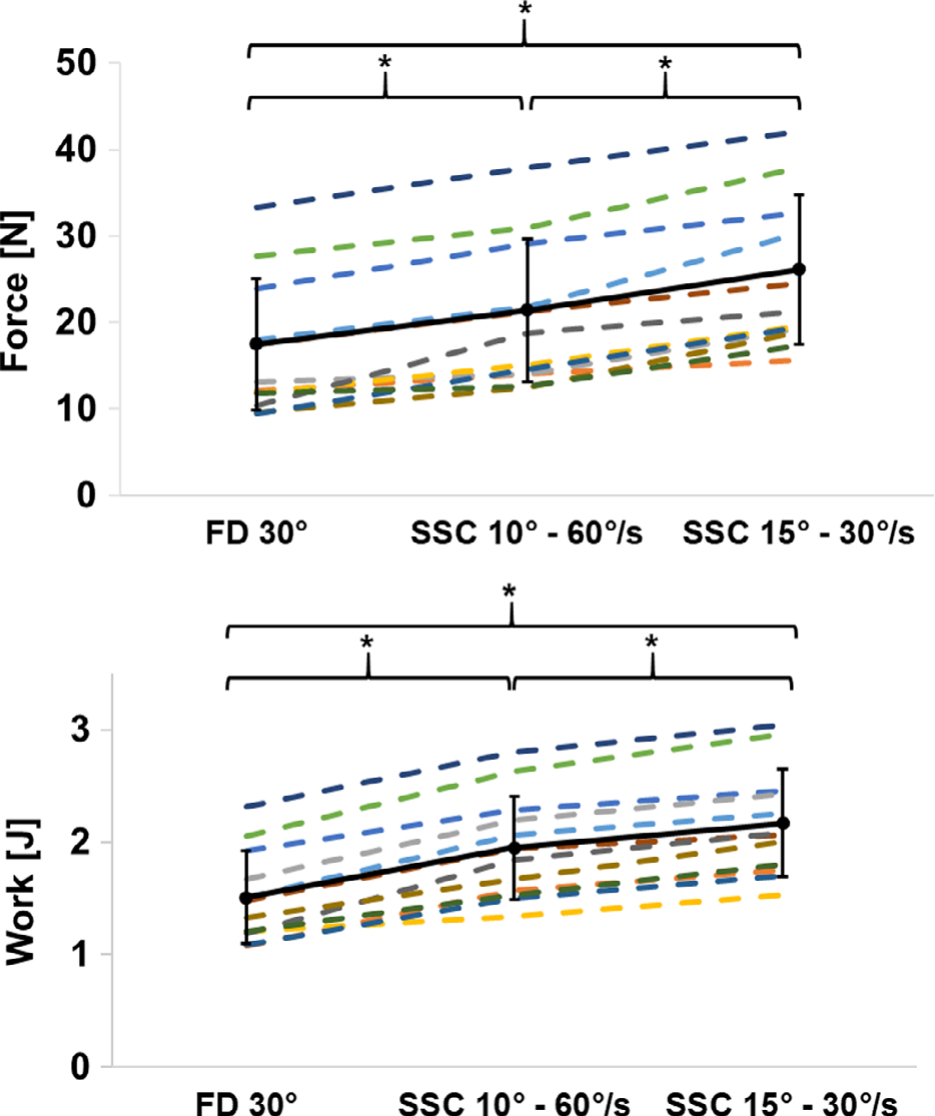
Mean (±SD) values of force at the end of shortening (e‐Sho; top) and mechanical work during shortening (bottom) for the pure shortening contraction (FD_30°_), SSC_10°–60°/sec_ and SSC_30°‐15°_ in protocol 2. The minimum force value at the end of shortening was significantly (*P* < 0.01) different across conditions. FD_30°_ showed the lowest minimum value of 16.6 ± 7.8 N, followed by SSC_10°–60°/sec_ and SSC_30°–15°_ (20.2 ± 8.3 N and 24.9 ± 8.7 N, respectively). The mechanical work during shortening was significantly different (*P* < 0.01) across conditions. SSC_30°–15°_ had the highest work produced with 2.17 ± 0.48 J, followed by SSC_10°–60°/sec_ and FD_30°_ (1.95 ± 0.46 J and 1.51 ± 0.41 J, respectively).

No significant differences (*t*(11) = −2.153, *P* = 0.054) were found for MTU forces at the end of stretch between SSC_10°–60°/sec_ and SSC_30°–15°/sec_ (79.5 ± 14.5N and 82.0 ± 13.9N, respectively), and forces were matched, by design, in the defined range of ±10% (Table 1). MTU forces at the end of shortening were significantly (*F* (2,22) = 89.16, *P* < 0.001) different between FD_30°_, SSC_30°–15°/sec_, and SSC_10°–60°/sec_. Post‐hoc tests showed significantly (*P* < 0.001) higher MTU forces for the SSC_30°–15°/sec_ (24.9 ± 8.7N), than the SSC_10°–60°/sec_ (20.2 ± 8.3N), and the pure shortening trials (FD_30°_, 16.6 ± 7.8N) (Fig. 4 and Table 1). ANOVA revealed significant (*F *(2,22) = 168.5, *P* < 0.001) differences between both SSCs and FD‐condition for mechanical work during MTU shortening. The mechanical work performed during MTU shortening was statistically (*P* < 0.001) the highest for SSC_30°–15°/sec_ (2.17 ± 0.48J), followed by SSC_10°–60°/sec_ (1.95 ± 0.46J), and FD_30°_ (1.51 ± 0.41J) according to Bonferroni‐Holm post‐hoc tests (Fig. 4 and Table 1).

Results of ANOVA showed significant (*F* (3,33) = 98.29, *P* < 0.001) differences between MTU isometric steady‐state forces between the MTU isometric reference, SSCs, and FD trials. The steady‐state MTU isometric forces were significantly (*P* < 0.001) depressed by −22.4 ± 5.8%, −19.3 ± 6.7%, and −13.0 ± 5.4% for FD_30°_, SSC_10°–60°/sec_, and SSC_30°–15°/sec_, respectively, compared to the isometric MTU reference contractions at the corresponding thumb angle. For FD_30°,_ there was a significant correlation between the amount of work during MTU shortening and force depression (*r_s_ = *0.67, *P* = 0.017). For SSC_10°–60°/sec_, and SSC_30°–15°/sec_ and the corresponding amount of rFD, no significant relationship was found (*r_s_ = *0.37, *P* = 0.24 and *r_s_ = *0.195, *P* = 0.54, respectively).

**Figure 5:**
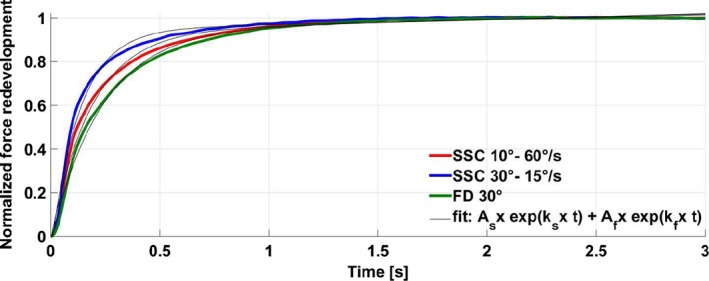
Exemplary data (*n* = 1) of force redevelopment and double exponential fit curves for FD_30°_, SSC_10°–60°/sec_, and SSC_30°–15°_. Force‐time data were normalized to the minimum force at the end of shortening (e‐Sho =0) and to the force in the isometric steady‐state 2.5–3 sec after shortening (=1). The time to redevelop 50% of steady‐state force after shortening termed half‐life period. SSC_30°–15°/sec_ had significantly (*P* < 0.01) the highest rate of fast force redevelopment (*k*f) followed by SSC_10°–60°/sec_ and FD_30°_ (−6.47 ± 1.72, −4.75 ± 1.11 and −3.99 ± 0.83, respectively). SSC_30°–15°/sec_ reached significantly (*P* < 0.01) faster 50% of the steady‐state force after shortening with a mean half‐life period of 122 ± 34 msec, followed by SSC_10°–60°/sec_ and FD_30°_ (164 ± 53 msec and 197 ± 51 msec, respectively).

MTU forces in the steady‐state phase for pure lengthening trials and the corresponding steady‐state forces following the stretch‐shortening tests showed strong correlations for the small and fast stretches (RFE_10°–60°/sec_ and SSC_10°–60°/sec_; *r_s_ = *0.64, *P* = 0.02605) and for the large and slow stretches (RFE_30°–15°/sec_ and SSC_30°–15°/sec_; *r_s_ = *0.65, *P* = 0.023). The rate of force redevelopment following active MTU shortening for the FD_30°_, SSC_30°–15°/sec_, and SSC_10°–60°/sec_ conditions could be approximated well (*R* ^2^ = 0.99) using equation (1) (Fig. 5). The rate of fast MTU force redevelopment (*k_f_*) after shortening was significantly different between all conditions (*F *(2,22) = 31.44, *P* < 0.001). Stretch‐shortening contractions (SSC_30°–15°/sec_ and SSC_10°–60°/sec_) showed faster rates of *k_f_* (−6.27 ± 1.78 and −4.62 ± 1.15, respectively) compared to pure shortening FD_30°_ (−3.85 ± 0.78) trials. There was no significant (*F* (2,22) = 1.077, *P* = 0.358) difference between conditions for slow MTU force redevelopment (*k_s_*) after shortening. The half‐life periods and the time needed to redevelop 50% of the steady‐state MTU force following the shortening phase were significantly (*F* (2,22) = 37.70, *P* < 0.001) different. FD_30°_ differed significantly (*P* = 0.002 and *P* < 0.001, respectively) from SSC_10°–60°/sec_ and SSC_30°–15°/sec_, and there was also a significant difference (*P* = 0.001) between SSC_10°–60°/sec_ and SSC_30°–15°/sec_. FD_30°_ showed the longest half‐life periods (203 ± 52 msec) followed by SSC_10°–60°/sec_ and SSC_30°–15°/sec_ (171 ± 55 msec and 127 ± 35 msec, respectively) (Fig. 5 and Table 2).

**Table 2:** Mean values (±SD) of force redevelopment rates and half‐life period according to equation (1) for FD_30°_, SSC_10°–60°/sec_, SSC_30°–15°/sec_. Parameters *k*_s_ and *k*_f_ show the rate of force redevelopment for the slow and fast phase after shortening. Half‐life period is the time needed to reach 50% of force in the steady‐state (* compared to FD_30°_. ^†^ compared to SSC_10°‐60°/sec_).

Contraction condition	*k_s_*		*k_f_*		Half‐life period [msec]	
	Mean	SD	Mean	SD	Mean	SD
FD_30°_	0.04	0.02	−3.85	0.78	203	52
SSC_10°–60°/sec_	0.04	0.01	−4.62^*^	1.15	171^*^	55
SSC_30°–15°/sec_	0.03	0.03	−6.27^*†^	1.78	127^*†^	35

## Discussion

The mechanisms behind history‐dependent forces in SSCs remain a matter of debate. Specifically, it remains unknown if and how rFE, produced during active muscle stretch, affects the forces in the subsequent shortening phase. We have shown previously that when rFE is kept constant, but the force at the end of the stretch is varied by changing the stretch speed, the forces at the end of the SSCs reached the same steady‐state isometric values (Fortuna et al. 2018). This result led to the hypothesis that the amount of rFE established during the stretch phase affects the shortening phase in SSCs, and not the force at the end of stretch, or the work performed during the shortening phase.

In order to test this hypothesis, we designed an experiment in which MTU rFE was systematically manipulated while the MTU force at the end of the stretch was kept constant. This was achieved by an intricate mixing of both the speed and magnitude of stretch. Briefly, the stretch magnitude was changed, thereby resulting in different rFE, while the stretch speed was adjusted such that a slow speed was used for the large stretch and a high speed for the short stretch until the forces at the end of the stretch were matched.

We hypothesized that rFE established during the stretch phase was the primary factor for changes in the steady‐state isometric MTU force following the SSCs. In agreement with this hypothesis we found that steady‐state force was greater following SSC conditions when rFE was greater, despite equal forces at the end of the stretch phase.

The present findings are in line with existing results describing rFE as long lasting (Herzog and Rassier 2002; Herzog et al. 2003), independent of the stretch speed (Abbott and Aubert 1952; Edman et al. 1982; Lee and Herzog 2002), but dependent on the stretch magnitude (Abbott and Aubert 1952; Edman et al. 1982; Lee and Herzog 2002). We found similar rFE values as Lee & Herzog (2002) for the same stretch magnitudes of the electrically stimulated adductor pollicis, confirming the dependence of MTU rFE on stretch magnitude. Residual force enhancement has been attributed to an active component (Herzog et al. 2006) (changes in cross‐bridge kinetics, either by increasing the proportion of attached cross‐bridges or an increase in the average force per attached cross‐bridge) and/or a passive component (Edman et al. 1982) (engagement of a passive element upon activation, most likely by the molecular spring titin, which may increase its spring stiffness (Labeit 2003; Joumaa et al. 2008b) or reduce its free spring length (Leonard and Herzog 2010; Powers  et al. 2014)) or the development of (half‐) sarcomere‐length non uniformities that develop upon active muscle stretching on the descending limb of the force‐length relationship. The results found here suggest that the amount of  rFE of the MTU established in the stretch phase of the SSCs affects the forces during the shortening phase of the SSCs, irrespective of force at the end of the stretch phase, and the forces in the final steady‐state isometric phase of our experiments. This suggestion is supported by the increased MTU forces at the end of shortening and in the final steady‐state isometric phase of the SSCs with greater rFE (after 30° stretch) compared to those with less rFE (after 10° stretch). We speculate that the rFE established during the stretch phase might be reduced by the immediate shortening following the stretch, maybe by mitigating the active component (cross‐bridge kinetics) of rFE. However, the passive component of rFE may carry over its effects into the shortening phase in a magnitude‐dependent manner.

### Transient force values

The MTU force values before (not shown) and at the end of stretching, and at the end of shortening were quantified to ensure a consistent force output among trials. Although we found a ~10% day‐to‐day difference in the absolute forces between protocols 1 and 2 in all variable measured (e.g., force values b‐str, e‐str, and e‐sho), this difference stayed constant for all normalized force parameters (e.g., rFE and rFD values normalized to the corresponding MVC) between Protocol 1 and 2. Thus, indicating high comparability of normalized data within the conditions and among the 2 days of testing. Forces at the end of stretch were matched in a ±10% range for SSC and RFE trials in Protocols 1 and 2 by manipulating the speed and magnitude of stretch individually for each participant.

Considering the force‐velocity (Hill, 1938) properties of skeletal muscles, we used a higher stretch speed for tests with the short stretch magnitude and vice versa to achieve matched force values at the end of the stretch phase of the SSCs. MTU force at the end of shortening in protocol 2 was significantly higher for SSCs compared to pure shortening contractions (FD_30°_). Specifically, the higher the amount of MTU rFE achieved during the stretch phase (SSC_30°–15°/sec_), the higher the force at the end of shortening. Thus, rFE of the MTU seems to directly counteract rFD in SSCs. As mentioned earlier, rFE is thought to incorporate an active (Abbott and Aubert 1952; Morgan et al. 2000) and a passive component (Herzog and Leonard 2002). Assuming that force depression is caused by an inhibition of cross‐bridge kinetics (Maréchal and Plaghki 1979), this would affect the active rFE component while an engagement of a passive element, possibly titin (Edman et al. 1982; Herzog and Leonard 2002; Powers et al.  2014; Herzog 2014bb), could retain its increased force during the shortening phase of SSCs.

### Work values

The mechanical work during shortening was significantly greater for all SSCs compared to the pure rFD shortening contractions, possibly as a result of significantly higher forces at the beginning of MTU shortening for the SSCs. Additionally, the higher the amount of rFE of the MTU  achieved during the stretch phase, the higher the amount of work performed during shortening. It is accepted that rFD is directly proportional to the amount of work during shortening for pure shortening tests (Herzog et al. 2000; Kosterina et al. 2009). A possible mechanism suggested for rFD following active shortening is a stress‐induced inhibition of cross‐bridge attachments that has been associated with the amount of mechanical work (Maréchal and Plaghki 1979; Joumaa et al. 2012). In this theory, it is assumed that increasing the stress on the actin filaments (by increasing muscular force and work) reduces the probability of cross‐bridge attachments. However, we found the opposite result for SSCs; increasing work was associated with decreasing rFD. The different amounts of work in the SSCs were not caused by different force values at the start of shortening/end of stretch, as these were matched on purpose through our experimental design. SSCs with the greatest amount of work during shortening and greatest force at the end of shortening had the least amount of rFD of the MTU. One possible explanation for the changed relationship between work and MTU rFD in SSCs compared to pure shortening contractions may be that in pure shortening contractions we would expect the work to be performed primarily by active cross‐bridge forces. While in SSCs, rFE likely contains a great passive component that would be expected to contribute substantially to the work in the shortening phase of SSCs (Herzog and Leonard 2002). It is accepted that rFD is caused by an inhibition of cross‐bridge attachments that is positively correlated with the active work produced by muscles in a pure shortening contraction (Maréchal and Plaghki 1979; Herzog et al. 2000; Joumaa et al. 2012). However, SSCs might not be affected in the same manner, as a substantial amount of the shortening work is expected to be produced by passive structural elements of the muscle‐tendon unit. In summary, this result might be caused by the fact that the shortening work in pure shortening contractions is primarily produced by active cross‐bridge forces, while much of the positive work in SSCs is likely caused by passive muscle elements (Edman et al. 1982; Powers et al. 2014; Herzog 2014bb).

### Force redevelopment

Corr & Herzog (2016) proposed that the rate of force redevelopment after shortening contractions decreases with increasing rFD and mechanical work during shortening indicating a stress‐induced inhibition of cross‐bridge attachments as the responsible mechanism for rFD (Corr and Herzog 2016; Fortuna et al. 2017). These findings are in contrast with our results and those of Seiberl et al. (2015) where a greater amount of work during shortening was related to faster force redevelopment and less rFD in the isometric MTU steady‐state for SSCs compared to pure MTU shortening tests. This result can also be explained readily with the idea that in pure shortening contractions, force redevelopment is caused by cross‐bridge attachments whose rates are slowed by stress on actin filaments. In the SSCs, force redevelopment is quicker since a substantial amount of stress is thought to be borne by passive structures and less by actin filaments, thus cross‐bridge attachment rates are less affected (i.e., quicker).

### Limitations

The present study has some limitations. First, it was difficult to perfectly match MTU forces at the end of the stretch phase for the combination of long stretch/slow speed and short stretch/high speed conditions. Therefore, we only used MTU forces at the end of the stretch that fell within 10% between conditions to be considered for comparisons. Although there was no statistical difference between MTU forces at the end of the stretch for the different conditions, by pure accident the majority of subjects (10 out of 12) showed slightly greater force at the end of stretch for the condition of long stretch. We showed previously (Fortuna et al. 2018) that there is no effect of force at the end of the stretch on the steady‐state force following the SSCs. However, rFE is known to be related to force at the end of the stretch in pure stretch trials (Bullimore et al. 2007) and some bias cannot be excluded. Additionally, there was a small (average of 2%), but significant difference in isometric MTU force at the beginning and at the end of protocol 2. However, since SSC trials with long and short stretch‐amplitudes were arranged in a randomized order, we believe that the results were not affected by fatigue.

## Conclusion

The steady‐state isometric MTU forces following SSCs seem to depend primarily on the amount of MTU rFE established in the initial stretch phase and appear to be independent of the force achieved at the end of the stretch phase. These results can be explained with the idea that there is an engagement of a passive structural element in the stretch phase of SSCs that does not exist in pure MTU shortening contractions. Experiments on the myofibrillar level, where the amount of titin and the actin‐myosin based cross‐bridge forces can be changed in a controlled manner, are needed to resolve these issues in an unequivocal manner.

## Conflict of Interest

The authors have no conflicts of interest to disclose.
